# Society Notes

**Published:** 1905-01

**Authors:** 


### Society Notes.

ANDERSON COUNTY MEDICAL SOCIETY met at Palestine, December 12th. No report. Retiring officers: J. M. Colley, President; G. R. Howard, Secretary. Full program.

BASTROP COUNTY MEDICAL SOCIETY.—Annual meeting, Bastrop, December 28th ult. Good attendance. Reception by Dr. and Mrs. H. P. Luckett. No report of meeting.

AUSTIN DISTRICT MEDICAL SOCIETY (Seventh Councilor District) met in Austin, December 22d ult., with large attendance and fine program. Dr. M. M. Smith, Austin, elected President; Dr. J. H. White, Kyle, Vice-President; Dr. W. A. Harper, Secretary (holds over).

LLANO-BURNET-MASON COUNTY MEDICAL SOCIETIES held joint session at Llano, December 28th. Fifty-three in attendance; swell twelve-course banquet at the Algona Hotel, Dr. C. F. Darnall, toastmaster. A splendid program was carried out; but we have not been favored with a full report of meeting.

THE EAST TEXAS MEDICO-CHIRURGICAL SOCIETY met at Crockett, December 12th. Dr. W. B. Collins, Jacksonville, elected President; Dr. F. L. Barnes, Trinity, Vice-President; Dr. J. B. Ramsay, Alto, Secretary and Treasurer. Banquet. Good meeting. Next meeting May, 1905, at Trinity, Texas.

TRAVIS COUNTY MEDICAL SOCIETY, annual meeting, Austin, December 15th. Officers elected: Dr. H. B. Hill, Austin, President; Dr. T. C. Pettway, Austin, Vice-President; Dr. H. F. Sterzing, Secretary. On Board of Censors: Dr. W. Neal Watt. Alternate delegate to State Medical Association, Houston, in April, Dr. W. J. Mathews.

TAYLOR COUNTY MEDICAL SOCIETY met at Abilene December 6th (ult.). Dr. J. B. Thomas, the Secretary, reports that the old officers were re-elected and all committeemen hold over. For January (3d) meeting they had: "Treatment of Epilepsy," Dr. J. H. Eastland; "Pneumonia," Dr. W. H. Barnett and W. D. Little. Discussion not reported.

THE BRAZOS VALLEY MEDICAL ASSOCIATION met in Franklin, November 22d, President, Dr. E. N. Shaw, of Cameron, in the chair. The following papers were read and discussed: "State and Local Quarantine Laws," Dr. Geo. R. Tabor, State Health Officer, Austin. "Malarial Hematuria," Dr. H. E. Baine, Lexington. "Medical Organization," Dr. Sam R. Burroughs, Buffalo. "Demonstrations on Hernia," Dr. J. E. Thompson, Professor of Surgery, Galveston Medical College. "Rupture of Uterus," Dr. W. A. Bedford, Franklin. "Animal Extracts," Dr. H. N. Graves, Georgetown.

A delightful smoker was given at the National Hotel on the evening of the 22d. The next meeting will take place at Cameron, on the second Tuesday and Wednesday in May, 1905. Thirty-three doctors were in attendance. The Robertson County Medical Association met with the B. V. M. A.

W. B. BRIGGS, Secretary-Treasurer.

SMITH COUNTY MEDICAL SOCIETY met at Tyler, December 13th ult. Secretary A. E. Woldert says: "Membership 32. Total number of physicians in the county 60; retired 7.

The Secretary announced that some of the physicians practicing medicine had not complied with all the requirements of the law. Their names will be furnished the Board of Censors, who may eventually turn same over to the grand jury. Every physician practicing medicine must comply with the law, or cease practicing.

Interesting addresses were made by Dr. T. J. Bell and Dr. D. H. Connally. The following officers were elected for the ensuing year: President, Dr. J. Charles Smith, of Starrville; Vice-President, Dr. R. H. Urquhart, of Tyler; Secretary and Treasurer, Dr. Albert Woldert, of Tyler (re-elected). Members of the Board of Censors who will compel physicians to comply with the medical law are Dr. T. J. Bell, Dr. J. D. Phillips and W. R. Johnson, of Winona. The Committee on Legislation appointed were Dr. Irvin Pope, Dr. A. L. Montgomery and Dr. T. J. Bell. The society then proceeded to election of delegates to State Medical Association which meets at Houston, Texas, in April, 1905. Dr. Albert Woldert was elected delegate and Dr. T. J. Bell alternate.

The Bell County Medical Society met at Temple, December 9th. Dr. J. M. Frazier, in his address as President, took for his subject, "Medical Co-operation and the Advantages of Medical Organization," which proved the writer thoroughly conversant with the needs of the profession along that line. Dr. W. L. Crosthwaite, of Holland, was elected President.

Since the organization of the society we have taken in fifty-four members, transferred three and had one death, which leaves a membership of fifty. This constitutes about 80 per cent of the legal practitioners in the county. The average attendance for all meetings is 46¼ per cent. After one of the Harvey House's famous suppers, given by the local physicians to their guests, the members gathered in the parlors of the hotel, where an interesting "social" was held.

Dr. W. L. Crosthwaite's paper on "The Code" was of exceptional interest. Taking the stand that a "written code" was necessary to standardize professional ethics, the doctor handled the subject in a masterly way. The next meeting will be held at Belton, the first Wednesday in March.

J. M. McCutchan, Secretary.

Bexar County Medical Society met in San Antonio December —, 1904, with a large attendance. They had, as usual, a swell banquet.

Dr. John S. Lankford presented a resolution urging upon physicians the extreme importance of reporting promptly to the health officer all cases of contagious and infectious diseases.

Dr. Marvin L. Graves introduced a resolution requesting the members of the society to familiarize themselves with the "anatomical bill," which will be presented before the next Legislature, so that some final action can be taken by the society at the January meeting. It was adopted.

The following officers were elected to serve during 1905: Dr. W. M. Wolff, President; Dr. Amos Graves, Jr., Vice-President; Dr. S. T. Lowry, Secretary; Dr. T. E. Moore, Treasurer; Dr. J. H. Burleson, Censor.

Dr. Fred Hadra officiated as toastmaster, and the following are some of the toasts: "Bexar County Society; Its Future," Dr. W. M. Wolff. "Experiences of a County Physician," Dr. D. Berrey. "The Army Doctor," Dr. W. P. Banta, U. S. A. "The Subjective Symptoms of a Doctor Who Handles Large Amounts of Money,"

THE ONE REMEDY

which experience proves is
free from detrimental effects is

GRAY'S Glycerine TONIC Comp.

Try it in convalescence,
respiratory disorders, anaemia,
malnutrition, nervous exhaustion.

THE PURDUE FREDERICK CO.,
298 Broadway, New York

Dr. W. L. Barker. "The Elixir of Life," Dr. J. V. Spring. "Diagnosis and Treatment of the Dead Beat," Dr. S. Burg. "The Etiology of Good Fellowship," Dr. T. T. Jackson. "The Annual Banquet," Dr. C. E. R. King. "Bacteriology from a French Standpoint," Dr. Charles A. R. Campbell. "The Bacillus of Love," Dr. B. Frank Stout. "The County Society Secretary," Dr. W. A. King, of Falls City. "Diplomacy," Dr. M. J. Bleim. "The Doctor in Politics," Dr. J. K. P. Green, of Rancho.

Other toasts were responded to by Drs. J. T. Beckmyer, of Hondo; Dr. T. B. Herbert, of Galt, Mo.; Dr. M. L. Graves, Dr. G. G. Watts and others.

